# 

**Published:** 1880-05

**Authors:** 


### THE AMERICAN JOURNAL OF DENTAL SCIENCE,

EDITED BY

F. J. S. GORGAS, M. D., D. D. S.,

AND

JAMES B. HODGKIN, D. D. S.

VOL. 14.—THIRD SERIES.

SURGEON GEN'LS OFFICE
82474
LIBRARY.

BALTIMORE:
SNOWDEN & COWMAN.
LONDON:
TRUBNER & CO., 60 PATERNOSTER ROW.
1880—1881.

### Index To Volume XIV.--Third Series.

Artificial Teeth, New Metho ls of Mounting, - - 1
A Valuable New Compound Metal, - - - 43
A New Disinfectant, - - - - 45
Anæsthesia Mesmeric, - - - - 47
Asbestos Powder as a Cement, - - - 48
An Anti-Scorbutic, - - - - - 48
Anæsthetics, New, - - - - 48
Advantages the American Colleges of Dentistry Afford the Student and Public, - - - - 53
Alveolar Abscess, - - - -- 58, 171
Aconitia in Neuralgia, - - - - 79
Arthur, Dr. Robert, Death of, - - - - 136
A Form of Land Scurvy, - - - - 143
A New Narcotic, - - - - - 144
Apparent Death as a Result of Asphyxia, - - 144
Amalgams, - - - - - 182
American Academy of Dental Science, - - 183, 426
Anæsthesia in Dentistry, - - - - 188
A New Anæsthetic, - - - - 189
Action of Collodion on Temperature, - - 190
American Dental Association, - - - - 191
Ataxia, - - - - - - 192
Anæsthesia by Ethyl Bromide, - - - 193
Arthur, Robert, M. D., D. D. S., - - - - 214
Administration of Chloroform, - - - 225
Advances in Medical Education, - - - - 235
A Method of Capping Exposed Pulps, - - 315
A Caution, - - - - - - 330
Another Discovery, - - - - - 332
Advances and Retrogression in Medical Teaching, - 572
Anæsthetics in 1651, - - - - - 335
Alloys and Solders, - - - - - 429
A Nerve Stimulant, - - - - - 141

Anæsthesia by Rapid Respiration, - - - 431
A Dentalation, - - - - - - 452
Address to the Graduating Class of the Balto. College of Dental Surgery of 1881, - - - 497
A Curious Fracture of Skull and Face, - - - 526
Adulterations in a New Sugar, - - - 528
American College of Dentistry, - - - - 53
At Last, - - - - - - 92
Articulating Artificial Teeth, - - - - 269
Apparent Death, - - - - - 286
A New Anæsthetic, - - - - - 287
Bromide of Ethyl, - - - - 76, 133, 266
Bromide of Ethyl as a Local Anæsthetic, - - 192
Bellevue Hospital Medical College, - - - 572
Bromide of Ethyl, Anæsthesia by, - - - 193
Brophy, Truman W., M. D., D. D. S., - - 17, 348, 470
Black, G. V., D. D. S., - - - - 289
Bleaching Gutta Percha, - - - - 576
Behaviour of Metals when Heated in Vacuo, - - 333
Board of Visitors of Balto. College of Dental Surgery, 476
Baltimore College of Dental Surgery, 41st Annual Commencement of - - - - 523

BIBLIOGRAPHICAL.

Homœopathy, - - - - 237
American Agriculturist, - - - 381
Scientific American, - - - - 381
Richardson's Mechanical Dentistry, - - 382
Atlas of Human Anatomy, - - - 382
Sanitarian, - - - - - 383
Walsh's Physicians Call Book, - - - 429
Websters' New Unabridged Dictionary, - 477
Transactions of Am. Med. Association 1880, - 477
Vicks Illustrated Guide, 1881, - - - 478
Horse Teeth, - - - - - 478
Transactions of Am. Dental Association 1880, - 478
Elementary Treatise on Practical Chemistry, and Qualitative Inorganic Analysis, - - 575
Essentials of Chemistry, - - - - 576
Journals, - - - - - 525

Nasal Catarrh, - - - - - - 281
Common Mind Troubles and the Secret of a
Clear Head, - - - - - 281
Report of Health Officers of the District of Columbia, 282
American Newspaper Directory, - - 282
The Skin in Health and Disease, - - 282
School and Industrial Hygiene, - - 283
Cement for Mending Hard Rubber, - - - 45
Compressed Nitrous Oxide, - - - - 44
Chloroform in Diseases of the Heart, - - 45
Cement for Rubber, - - - - 48
Cure of a Traumatic Salivary Fistula, - - 94
Call for a Convention to Organize a National Dental
Association, - - - - - 131
Chloroform in Diseases of the Heart, - - - 142
Coyle, John H., D. D. S., - - - - 176
Compressed Mother of Pearl, - - - - 188
Changes in the Maxillary Bones in Ataxia, - - 192
Chloroform, Administration of, - - - 225
Chancres of the Tonsil and Mouth, - - - 239
Caries of the Teeth, Natural History of, - - 289
Capping Exposed Pulps, - - - - 315, 379
Carbolic Acid and Creasote, - - - - 348
Codman, John T., D. D. S., - - - - - 363
Celluloid Infringement, - - - - - 379
Carbolic Acid, Toxic Effects of, - - - 383
Carbolic Acid Poisoning, - - - - 384
Correspondence between Dr. T. B. Gunning and the
Census Bureau, - - - - - 400
Cervico-Bronchial Neuralgia Cured by the Extraction
of Wisdom Teeth, - - - - - 431
Chloroform, Dangers of, - - - - - 432
Clowes, J. Washington, D. D. S., - - - - 452
Caries of the Superior Maxilla, - - - - 470
Cutter, Ephraim, M. D., - - - - - 504
Cohesive and Non-Cohesive Golds, - - - 518
Curious Fracture of Skull and Face, - - - 526
Capping Exposed Pulps, - - - - - 82
Classic Dentistry, - - - - - - 288
Dental Hemorrhage, - - - - - 47

Death of the Dental Pulp by External Violence, - 31
Disinfectant, A New, - - - - - - 45
Dop, Dr. G., - - - - - - 31
Dental Caries, - - - - - - - 68
Death from Bromide of Ethyl, - - - 191
Diagnosis of Malignant Tumors of Upper Jaw, - - 206
Diploma Selling, History of, - - - 220
Dental Surgery in the United States, - - - 308
Dental Decay, Prevention of, - - - 325
Disturbances of Vision by Unsound Meat, - - 333
Diamond Instruments, - - - - 428
Driscoll, W. E , D. D. S., - - - - 269
Diseases of Wild Animals. - - - - 272
Extraction of First Molars, - - - - 38
Effects of Smoking on the Teeth, - - - 47, 83
Ebert, Dr. Louis R., - - - - - 68
Electro-Chemical Theory, - - - - 166
Ethics of Dentistry, - - - - - 176
Essay on Mechanical Dentistry, - - - 145
Effects of Smoking on the Heart, - - - 240
Forty-first Annual Course of Instruction in the Baltimore
College of Dental Surgery, - - - 279
Farrar, J. N., M. D., D. D. S., - - - 241
False Teeth, - - - - - 184
Flagg, E. M., D. D. S., - - - - 357
Filling Teeth, Some Special Points to be Observed, - 337
Faught, L. Ashley, D. D. S., - - - - 510
Fox, Francis, - - - - - 419
Ford, Dr. W. W., - - - - - 145
Fletcher, Thomas, - - - - 182
Fetid Sweating of the Feet, - - - - 432
Finley, M. F., D. D. S., - - - - - 58
Fatal Carelessness, - - - - - 384
Feeding of Nursing Children, - - - - 504
General Care of the Mouth of Patients under Twenty
Years of Age, - - - - - 259
Goodyear Dental Vulcanite Company, - - 427
Goodyear Patent, - - - - - 384
Gunning, Thomas B., D. D. S., - - - - 400
Glass Wicks for Lamps, - - - - 335

Harlan, A. W., D. D. S., - - - - - 38
History of Diploma Selling, - - - 220
Homœopathy, - - - - - - 237
Hodgkin, Prof. Jas. B., D. D. S., - - - 442
Howard, F. E., D. D. S., - - - - 460
Heveenoid. The Rubber of the Future, - - 513
Is it Found? "Naboli," - - - - - 89
Is Expansion a Cause of Leakage in Fillings, - - 91
Important Notice, - - - - - 93
Influence of Tobacco Smoking upon Health, - - 186
Irregularities of the Teeth and their Surgical Treatment, 419
India Rubber Deterioration, - - - - 480
Importance of Direct Sunlight in the Operating Room, 241
Is Salicylic Acid Injurious to the Teeth, - - 288
Jobson, M. S., D. D. S., - - - - - 315
Keeping Rubber Plates Clean, - - - 432
Kirkus, Rev. William, M. A., LL. B., - - - 479
Koch, C. R. E., D. D. S., - - - - - 171
Landesberg, M., M. D., - - - - - 377
Mechanical Dentistry Items, - - - 41
Mechanical Dentistry, - - - - 145, 357
Manufacture of Celluloid, - - - - 47
McDonald, Dr. Geo. B., - - - - - 63
Memorial of Herr Theodore Melhardt, - - - 49
More About Rigg's Disease, - - - - 160
Mills, Dr. Geo. A., - - - - - 160
Macdonnel, Richard L., M. D., - - - 225
Medical Education, - - - - - 235
Method of Capping Exposed Pulps, - - 315
Maryland and District of Columbia Dental
Association, - - - 329, 385, 433, 481, 530
Motion in Teeth, - - - - - - 336
Maryland Dentists and the Vulcanite Patent, - 427
Mott, Prof. Henry A., Jr., - - - - 513
New Method of Mounting Artificial Teeth, - - 1
Neuralgia and its Treatment, - - - - 17
New Compound Metal, - - - - - 43
Necrosis, - - - - - - - 72
Natural Dentine for Capping Exposed Pulps, - - 82
Neuralgia, Treatment of, - - - - 85

National Dental Association, - - - 134
Nitro-Glycerine, - - - - - 336
Neuralgia of the Fifth Pair Cured by Aconite, - 377
Noyes, E., D. D. S., - - - - - 337
OBITUARY.
Dr. S. P. Cutler.
Dr. J. C. Harris,
Thomas Bell, F. R. S., Etc.,
Robert Arthur, M. D., D. D. S.
Operative Dentistry. - - - - - 125
Ohio State Dental Society, - - - - 425
Obermuller, C. H. E., D. D. S., - - - - 49
Odontological Society of Great Britain, - - 97
Our Dental Literature, - - - - 510
Proof of Death, - - - - - 44
Precautions in Anæsthesia, - - - - 46
Proper Management of Proximate Cutting and Grinding Surfaces of the Teeth, - - - - 63
Palmer, S. B., D. D. S., - - - - - 166
Progress of Dental Surgery in the United States, - 308
Patrick, J. B., D. D. S., - - - - 308
Patrick, C. C., D. D. S.. - - - - 325
Prophylaxis or Prevention of Dental Decay, - 325
Preservation of all the Natural Teeth, - - 363
Physical Examination of Mouth and Throat—The Physical Signs Derived from the Breath, Lips, Teeth and Mouth, - - - - - 367
Poore, G. V., M. D., F. R. C. S., - - - 367
Pulp Capping, - - - - - 379
Pathology and Therapeutics from a Conservative "New Departure" Standpoint, - - - - 460
Protective Legislation for Dentistry, - - 475
Poisoning by Tincture of Arnica, - - - 480
Pivot Teeth, - - - - - 490
Publishers of American Journal of Dental Science, 524
Position of the Dentist, - - - - 277
Philadelphia Bogus Diplomas, - - - 543
Researches on Hearing through the Medium of the Teeth and Cranial Bones, - - - - 9
Rubber Cement, - - - - - 48

Rudolph, Dr. F. W., - - - - - 53
Recent Dental Conventions, - - - - 185
Ready Way to test Chloroform, - - - - 189
Remedy in Thrush, - - - - - 190
Restoration of Contour as a Means of Separating Enamel Margins and Preventing Recurrence of Decay, - 392
Rapid Breathing as a Pain Obtunder, - - 275
Some Points in the Natural History of Caries of the Teeth and the Value of Fillings for its Arrest, - - 289
Safety in Hydrobromic Ether, - - - 191
Stellwagon, Thomas C., M. D., D. D. S., - - - 82
Source of Muscular Power, - - - - 46
Supression of Fraudulent Diplomas, - - - 87
Setting of Plaster. - - - - - - 143
Socket Instruments, - - - - - 330
Spence's Metal, - - - - - 334
Swallowing False Teeth, - - - - 287
Steel, Charles L., D. D. S., - - - - 490
Tiffany, L. Mc Lane, M. D., - - - - 206
Thackston, W. W. H., M. D., D. D. S., - - 214
Tasting Power of the Tongue, - - - - 430
Taylor, L. C., D. D. S., - - - - 259
Texas State Dental Association, - - - 520
Therapeutical Effects of Chlorate of Potash, - 527
Tragedy in a Dental Office, - - - - - 285
Toxic Effects of Carbolic Acid, - - - - 383
Temperature of the Breath, - - - - - 288
Tooth Structure Illustrated, - - - - 479
The Late Session of the Balto. College of Dental Surgery, 523
The Proposed extension of the Cumming's Patent, - 473
The Celluloid Infringement, - - - 379
The Fraudulent Diploma Man, - - - 380
To Our Young Men—A Caution, - - - - 330
The Value of Prolonged Artificial Respiration, - 335
Treatment of Teeth with Dead Pulps and Alveolar Abscess, 171
The Bromide of Ethyl as a Local Anæsthetic, - - 192
Thrush, Remedy for, - - - - - 190
Thomas, Charles Herman M. D., - - - 9
Treatment of Salivary Fistula, - - - - - 46
To Check Dental Hemorrhage, - - - - 47

Use and Abuse of Tobacco, - - - - - 95
Use of Chloroform in Angina Pectoris, - - 142
Use of Chloroform in Diseases of the Heart, - - 285
Unerupted Teeth in the Roof of the Mouth, - - 331
Value of Revaccination, - - - - 96
Value of Prolonged Artificial Respiration, - - 335
Vilian, Prof. Jean, - - - - - 272
Vulcanite Company vs. Celluloid Company, - - 410
Vulcanizer Explosions, - - - - 320
Virginia State Dental Association, - - - 183
Washing Amalgams, - - - - 182
Wax Free, - - - - - - 479
Watching the Pulse during the Administration of
Chloroform, - - - - - 283
Webb, Marshall, D. D. S., - - - - 392
Wilson, H. A., M. D., - - - - - 193
What Others Say, - - - - - 233
Wetherbee, Isaac J., D. D. S. - - - 125

### OBITUARY.

At a called meeting of the Dentists, and the "Microscopic Society" of Memphis, Tennessee, the following resolution was passed unanimously:

*Resolved,* That we announce with great regret the death of Drs. S. P. Cutler and J. C. Harris, of this city, and desire to record our testimony to their sterling worth as men and citizens, whose vast knowledge, ceaseless energies and probity of character were exponents of, and gave dignity to the dental profession; and to convey to their bereaved families the assurance of our most sincere condolence.

J. L. Newborn, *Secretary.*

### To Readers and Correspondents.

Communications intended for publication, and exchange journals and papers, should be addressed to the EDITOR OF THE AMERICAN JOURNAL OF DENTAL SCIENCE, 259 N. Eutaw St., Baltimore, Md. All letters relating to the business of the journal, or containing cash remittances, to be directed to Messrs. SNOWDEN & COWMAN. Articles for publication should be received by the editor at least three weeks previous to the first day of the month on which the number in which it is designed for them to appear, is issued.

### CONTENTS:

### MONTHLY SUMMARY.

Proof of Death...................................................44

Compressed Nitrous Oxide as an Anæsthetic.......................44

A New Disinfectant...............................................45

Chloroform in Diseases of the Heart..............................45

Cement for Mending Hard Rubber...................................45

Treatment of Salivary Fistula....................................46

Source of Muscular Power.........................................46

Precautions in Anæsthesia........................................46

Mesmeric Anæsthesia..............................................47

Manufacture of Celluloid.........................................47

To Check Dental Hemorrhage.......................................47

The Effects of Smoking upon the Teeth............................47

Asbestos Powder as a Cement......................................48

An Anti-Scorbutic................................................48

The New Anæsthetics..............................................48

Rubber Cement....................................................48

### OBITUARY.

Drs. S. P. Cutter and J. C. Harris...............................48

☞ The American Journal of Dental Science will contain fifty pages of reading matter in each number, making a volume of about

*Six Hundred* pages

EXCLUSIVE OF ADVERTISEMENTS.

THE

### AMERICAN JOURNAL

OF

### DENTAL SCIENCE.

The *Journal* is issued on the first of each month and contains not less than *fifty* pages of reading matter in each number, exclusive of advertisements, making a volume of *six hundred* pages per annum.

In each number will be found Original Communications, Correspondence, Selected Articles, Monthly Summary, Bibliographical Notices and Editorial Matter, and every effort will be exerted to render the *Journal* worthy of the patronage of the Dental profession.

A generous co-operation is respectfully solicited from the members of the profession; and as the *Journal* has now a printing office of its own, which materially lessens the cost of its publication, it will be issued at the reduced subscription price of

**$2.50 Per Annum in Advance.**

Communications are respectfully solicited on all subjects pertaining to the practice of dentistry, as well as reports of cases occurring in dental practice.

### RATES OF ADVERTISING.

| | 1 MO. | 2 MO. | 3 MO. | 6 MO. | 12 MO. |
|---|---|---|---|---|---|
| 1 PAGE. | $15 00 | $22 50 | $30 00 | $50 00 | $100 00 |
| ½ " | 10 00 | 15 00 | 20 00 | 30 00 | 50 00 |
| ¼ " | 7 00 | 11 00 | 15 00 | 20 00 | 30 00 |
| ⅛ " | 5 00 | 8 00 | 11 00 | 15 00 | 20 00 |

All original communications designed for publication, works for review, new instruments for notice, exchanges, &c., should be directed to the editor, 259 N. Eutaw St., Baltimore, Md.

All advertisements and subscriptions should be addressed to

SNOWDEN & COWMAN,

*Publishers of the Am. Journal of Dental Science.*

86 W. FAYETTE ST. BET. CHARLES & LIBERTY STS.,

BALTIMORE, MD

